# Snacktivity™ to Promote Physical Activity: a Qualitative Study

**DOI:** 10.1007/s12529-021-10040-y

**Published:** 2021-11-15

**Authors:** Natalie Tyldesley-Marshall, Sheila M. Greenfield, Helen M. Parretti, Kajal Gokal, Colin Greaves, Kate Jolly, Ralph Maddison, Amanda J. Daley, Snacktivity™ Study Team, Stuart Biddle, Charlotte Edwardson, Dale Esliger, Emma Frew, Natalie Ives, Nanette Mutrie, James Sanders, Lauren Sherar, Magdalena Skrybrant, Tom Yates

**Affiliations:** 1 School of Sport, Exercise and Health Sciences, Loughborough University, Loughborough, LE11 3TU Leicestershire UK; 2 Institute for Applied Health Research, School of Medical and Dental Sciences, University of Birmingham, Edgbaston, Birmingham, B15 2TT UK; 3 Norwich Medical School, University of East Anglia, Norwich, Norfolk, NR4 7TJ UK; 4 The Centre for Lifestyle Medicine and Behaviour (CLiMB), School of Sport, Exercise and Health Sciences, Loughborough University, Loughborough, LE11 3TU Leicestershire UK; 5 School of Sport and Exercise Sciences, University of Birmingham, Birmingham, B15 2TT UK; 6 Institute for Physical Activity and Nutrition, Deakin University, Melbourne, Australia

**Keywords:** Snacktivity™, Small bouts, Physical activity, Qualitative research, Interviews

## Abstract

**Background:**

Adults should achieve a minimum of 150 min of moderate-to-vigorous intensity physical activity per week, but many people do not achieve this. Changes to international guidance have removed the requirement to complete physical activity in bouts of at least 10 min. Snacktivity is a novel and complementary approach that could motivate people to be physically active. It focuses on promoting shorter (2–5 min) and more frequent bouts, or ‘snacks’ of physical activity throughout the day. It is not known whether promoting physical activity in shorter bouts is acceptable to the public, or whether it likely to translate into health behaviour change.

**Methods:**

As part of a larger research programme, this study explored the merits of using small bouts of physical activity to help the public become physically active (the Snacktivity™ programme). Thirty-one inactive adults used the approach for five days then participated in semi- structured interviews about their experiences. The data were analysed using the Framework approach.

**Results:**

Whilst participants highlighted some potential barriers to implementation, they expressed the ease with which Snacktivity could be achieved, which gave them a new awareness of opportunities to do more physical activity throughout the day. Participants raised the importance of habit formation to achieve regular small bouts of physical activity.

**Conclusions:**

Findings demonstrated that participants liked the Snacktivity concept and viewed it as a motivating approach. Guidance about physical activity must lead to advice that has the best chance of preserving and promoting health and Snacktivity has potential to meet this ambition.

**Supplementary Information:**

The online version contains supplementary material available at 10.1007/s12529-021-10040-y.

## Introduction

Guidance for participation in physical activity in many countries states that over a week, aerobic-based physical activity should accumulate to a minimum of 150 min of moderate-vigorous intensity physical activity (MVPA) [1], frequently promoted as a goal of 30 min on at least 5 days per week [2]. Meeting this guidance however often means making large behavioural changes, and many in the population do not achieve the recommended levels [2]. Given the undisputed health benefits from all types of physical activity, encouraging people to be active is a key objective of public health initiatives [1, 3]. However, if this is to be achieved, a shift in emphasis in the way physical activity is promoted is needed.

Whilst guidelines provide information on the minimum level of physical activity required for health benefits, and are useful for surveillance, intervention planning, and policy, they are not designed to motivate individuals to adhere to physical activity. There have been calls for physical activity guidelines to be translated into messages that are informative, provocative, persuasive, and which influence factors that determine physical activity behaviour (e.g., motivation and feelings of competence) [4]. Messaging for initiating behaviour change needs to straddle the desire to raise awareness of the importance of physical activity, whilst also being innovative, attractive, and motivating to the public.

### Snacktivity to Promote Physical Activity

Internationally, guidance now recognises that some physical activity is better than none, with the necessity for physical activity to be achieved in at least bouts of 10-min duration now removed, although there is still a focus on the public needing to achieve a large behavioural physical activity goal of at least 150 min of MVPA per week [1–5]. Advising the public to just ‘move more’, without providing a framework, or a means to achieve this, is unlikely to be successful. Individuals will require support to translate their intentions into action. Guidance that could motivate the public to be more active and also break up sedentary behaviour is a concept we have called Snacktivity™, which promotes short ‘snack’ size bouts of MVPA throughout the whole day, to progress towards meeting the guidance target of 150 min of MVPA per week. A physical activity snack would typically last 2–5 min, and examples might include brisk walking during a coffee breaks, walking when using a mobile phone, dancing while cooking, calf raises while teeth brushing, and leg raises when watching television. Crucially, such activity snacks can be integrated with other activities, so they do not necessarily require additional time. See supplementary file 1 for examples of Snacktivity.

The idea that small bouts, or accrued physical activity, may improve health outcomes is not new, as tested in laboratory or experimental studies [6–10]. However, it is not a message that has been prominently and consistently conveyed to the public because there is lack of high-quality evidence in real-world settings that it leads to sustained physical activity behaviour change in the population. At the heart of the public health guidance for physical activity and accompanying infographics, there is typically an emphasis on the public achieving 150 min of MVPA per week. The focus of physical activity strategies must be on removing barriers to participation, rather than simply trying to convince the public of the health benefits. In inactive adults (who are of most concern), the Snacktivity message may help to develop their confidence to become physically active, a strong predictor of adherence [11]. Psychological theory and perspectives acknowledge that achieving small changes can be important for developing feelings of self-efficacy (task and self-regulatory), a key technique for promoting changes in physical activity [12–14]. Simple or small actions may become routine more quickly than complex ones, highlighting that the integration of ‘snacks’ of physical activity within everyday life may be viewed as more feasible for people to initiate and then maintain, than trying to achieve large(*r*) changes [15].

A common reason for physical inactivity is a perceived lack of time [16]; Snacktivity can be framed as only requiring a few minutes at a time and is able to be integrated into existing activities or daily tasks. The Snacktivity message may be particularly appropriate for specific populations such as those with chronic diseases or disabilities, who may find shorter ‘snacks’ of physical activity more accessible and easier to achieve. Of additional concern is that adults spend a substantial part of their waking day (60–70% of hours) sedentary; Snacktivity provides opportunities to break up daily sitting time [17]. Guidance also recommends that the public should participate in muscle-based activities for increasing strength at least twice per week, and many of these types of activities can be achieved through short, frequent bouts during the day (e.g., seated leg raises & squats [1, 5].

### Aims of the Study

To support the development and future implementation of Snacktivity, this study explored the views of the public about the Snacktivity concept, and whether it is an approach that is likely to be acceptable. This study also examined potential barriers to Snacktivity and explored ways the message of ‘Snacktivity to increase physical activity’ could be refined and best promoted to the public.

## Methods

### Paradigm and Research Design

Epistemologically, the present study followed a constructivist paradigm that aligned with its descriptive and exploratory objectives [18]. This paradigm considers that individuals develop a subjective meaning of their experience that is dependent on setting, history, and cultural norms [18]. The study was underpinned by a relativist ontology that recognised that there is no reality independent of perception, and reality is multiple, created, and mind-dependent [19], and it was considered that these would align well with the aims of the study. This study is part of a larger programme of research that aims to explore and investigate the benefits of Snacktivity for health (https://fundingawards.nihr.ac.uk/award/RP-PG-0618-20008). Focus groups were initially planned for data collection but due to COVID-19 individual telephone interviews were conducted [20]. The Consolidated Criteria for Reporting Qualitative Research was used for reporting this study [21]. A team of 10 public advisory group (PAG) members have contributed their experience to the design and development of this study, reviewing all study documents, including the participant information sheet and patient consent form. The study was granted favourable ethical opinion by the East Midlands Leicester South Research Ethics Committee (19/EM/0370).

### Participants

The primary purpose of Snacktivity is to increase physical activity in people who are not already physically active. As such, physically inactive adults who had completed a prior Snacktivity survey were recruited from six diverse general practices in the West Midlands, England (Table 1). To increase the study breadth, the sample was supplemented by inactive employees from a local National Health Service (NHS) Trust who had also completed the prior Snacktivity survey (Table 1). Purposive sampling was used to invite ‘inactive participants’ (identified using survey responses to the General Practice Physical Activity Questionnaire (GPPAQ) [22] (as part of a prior Snacktivity study survey) who had also indicated interest in participating in further Snacktivity studies when completing the prior Snacktivity survey. Physically inactive status was defined as being unemployed, or in a sedentary job and completing less than 1 h of physical activity per week. Participants were invited to complete an interview in order of their returned survey.

**Table 1:** Participant characteristics

**No**	**Age category**	**Gender**	**Ethnicity**	GP Practice IMD*	IMD*
PI1	61–70	Female	White	5	20% most deprived
PI2	61–70	Male	White	1	10% most deprived
PI3	61–70	Male	White	9	20% least deprived
PI4	21–30	Female	Black other	10	40% most deprived
PI5	61–70	Male	White	9	10% most deprived
PI6	51–60	Male	White	9	20% least deprived
PI7	61–70	Male	White	9	20% least deprived
PI8	21–30	Female	Indian	9	40% least deprived
PI9	> 71	Female	White	5	50% most deprived
PI10	51–60	Female	White	5	20% most deprived
PI11	41–50	Female	White	10	10% least deprived
PI12	61–70	Female	White	1	10% most deprived
PI13	61–70	Male	White	10	10% least deprived
PI14	61–70	Male	White	1	20% most deprived
PI15	31–40	Male	Pakistani	1	20% most deprived
PI16	> 71	Male	White	10	10% least deprived
PI17	> 71	Male	White	10	10% least deprived
PI18	61–70	Male	White	10	10% least deprived
PI19	51–60	Female	White	10	10% least deprived
PI20	51–60	Female	White	10	50% least deprived
PI21	> 71	Female	White	10	20% least deprived
PI22	51–60	Female	White	1	20% most deprived
PI23	61–70	Female	White	10	10% least deprived
PI24	61–70	Female	White	10	10% least deprived
PI25	> 71	Female	White	10	10% least deprived
PI26	61–70	Female	Black Caribbean	1	10% most deprived
PI27	61–70	Female	White	10	10% least deprived
SI1	41–50	Female	Other Asian	N/A	30% most deprived
SI2	21–30	Female	Black African	N/A	10% most deprived
SI3	41–50	Female	Indian	N/A	Not provided
SI4	41–50	Female	Pakistani	N/A	Not provided

^*^The Indices of Deprivation are a unique measure of relative deprivation at a small local area level across England. The Indices provide a set of relative measures of deprivation across England, based on seven different domains of deprivation; income; employment; education, skills, and training; health and disability; crime; and barriers to housing and services and living environment. Combining information from the seven domains produces an overall relative measure of Index of Multiple Deprivation. P identification codes refer to patients, and S codes refer to NHS employees

### Data Collection

Physically inactive survey respondents who had indicated they were interested in taking part in further studies about promoting short bouts of physical activity (Snacktivity) were sent the participant information sheet and provided either verbal or written informed consent prior to their interview. As the purpose of the study was to understand people’s views about Snacktivity within their everyday life, participants were given a picture board leaflet illustrating different examples of Snacktivity (see Supplementary file 1) and asked to incorporate Snacktivity into their lives for at least five days prior to their interview. As Snacktivity is aimed at people who are inactive, and who will meet the MVPA threshold more quickly than those who are already physically active (at least initially), the picture board included activity snacks that were mostly light to moderate intensity, but which could be adapted to increased intensity as participants progressed in their ability and confidence. Some light and vigorous activity snacks were also included to ensure the picture board examples were suitable for a range of physical abilities and preferences. In line with physical activity guidance, strength-based activity snacks were also included.

The main interview topics of conversation were consistent with the aims of the study. Semi-structured interviews allowed the order and wording of the interview questions to be adapted as needed in the interviews, and additional questions asked to explore unexpected topics [23]. Interviews were undertaken by a female researcher (NTM) with many years’ experience in qualitative research methods and master’s degree in social science. Field notes of researcher reflections and initial thoughts around findings were written up immediately after each interview. The interviews were conducted in English and recorded using a password encrypted dictaphone, professionally transcribed and field notes integrated into transcripts. Participants received a £10 shopping voucher for participating. Socio-demographic data were collected in the prior survey.

### Data Analysis

Thematic analysis was supported by the principles of the Framework Method. The lead researcher (NTM) checked and then became familiar with the transcripts, inductively coding line-by-line [24]. An early transcript was independently reviewed by four authors (NTM, SG, AD, HP) with different backgrounds (sociology, psychology, general practice). Codes were then discussed, agreed, grouped together into themes, and thereafter a ‘framework’ agreed, which was applied to and refined by subsequent interviews. Interpreting the data and searching for patterns was undertaken throughout [24]. When later interviews (after 22 had been completed) did not contribute any new information to the story or ‘theory’, we judged that we had reached data saturation [25]. We continued until 31 interviews had been completed to ensure we were able to interview sufficient participants from non-White ethnic backgrounds. When the themes were finalised, the lead researcher entered the data into a framework matrix and then summarised. This step distinguishes the Framework Method from more standard thematic analysis and provides transparency in the coding and analysis process [24]. Data analysis was supported by NVivo 12 Plus [26].

## Results

### Participants

A total of 559 Snacktivity surveys were returned by patient participants and of these 292 expressed an interest in taking part in future Snacktivity research such as this qualitative study, of whom 68 were eligible and invited to take part here; 32 did not respond to this invitation, nine declined, and 27 consented and were interviewed. Additionally, a total of 166 complete surveys were returned by NHS staff and of these 111 expressed an interest in participating in future Snacktivity research; five were eligible and invited to take part, one declined, and four consented and were interviewed. Interviews lasted between 22 and 89 min. Patient participant interviews took place May–September 2020 and NHS staff interviews March–April 2020. See Table 1 for participant characteristics.

### Themes

The main themes that emerged from the data were types of activity snacks completed, Snacktivity as concept (including feelings about Snacktivity, likes and dislikes, perceptions of other people’s feelings about Snacktivity, suggestions for improving the Snacktivity concept), barriers to physical activity, and facilitators to physical activity.

### Which Activity Snacks Did Participants Do?

Most participants had incorporated extra walking-based activity snacks, either at a brisk or low intensity. See Table 2 for a detailed list. Most participants reported they had engaged in the following types of Snacktivity; stair climbing/descending, housework, walking while on their telephone, and calf raises while washing up.

**Table 2:** Number of participants engaging in Snacktivity

Snack no	Snacktivity description	Number of participants (*n* = 31)
**While at work**		
*9*	Arm raises while seated	12
*8*	Park your car further away and walk	6
*6*	Use the toilet that is furthest away/on another floor	4
*7*	Walking meeting with colleagues	4
*2*	Move away from your desk and walk while making calls	3
*3*	Brisk walk at lunchtime	3
*1*	Take the stairs instead of the lift or escalators	3
*10*	Walk over to talk to colleagues instead of using the phone or email	3
*4*	Lunges at work	2
*5*	Get off the bus one stop earlier and walk	1
**At home and in leisure time**		
*14*	Walk up and down the stairs multiple times	26
*15*	Housework	24
*19*	Walking whilst talking on the phone	23
*22*	Calf raises while washing the dishes	22
*13*	Moving whilst you wait for the kettle to boil	20
*28*	March on the spot	19
*29*	Gardening	17
*11*	Squats whilst brushing your teeth	17
*12*	Press-ups against the stairs	14
*17*	Lunges whilst you vacuum the house	13
*20*	Use a basket whilst shopping instead of a trolley	13
*21*	Walk/run/cycle to the local shops	13
*18*	Biceps curls with tins/a bottle while seated	12
*30*	Dance around the living room/kitchen	11
*25*	Brisk walk around your local park	8
*24*	Take dog for an extra brisk walk	7
*23*	Play with your children at the park	4
*27*	Skipping	4
*16*	Wash your car	3
*26*	Walk to drop off/pick your children up from school	0

### Snacktivity as a Concept

#### Bites of Physical Activity

Almost all participants understood that Snacktivity was about incorporating physical activity into their everyday lives and understood Snacktivity to mean short periods of activity or exercise like a ‘snack’ or ‘bite’ of physical activity, rather than ‘a main course’. Participants talked about the idea that Snacktivity allowed them to feel a sense of achievement because it was easy (or easier) to achieve. Some participants mentioned they understood the purpose of their activity snacks was to work towards at least 150 min MVPA per week.

P: [Snacktivity] means doing physical activity in all bits and pieces during the day and making them intrude minimally, minimally shall we say? On the rest of your day. Patient 16

I think of it [Snacktivity] as being…. a way of trying to… increase the amount of exercise you do but in… rather than in large chunks, rather than having … not like having a meal, you’re just having like a biscuit! Patient 18

#### The Flexibility of Snacktivity

Participants viewed Snacktivity as flexible for them in some way, with half commenting on the wide range of activity snacks available to choose from, with varying levels of difficulty. About half of participants discussed that Snacktivity could also be tailored to people’s individual circumstances, and it was suitable for people of all ages, levels of physical fitness, and those with medical conditions. Some participants mentioned that Snacktivity could be completed at any time of day, and that no special equipment, or a change of clothing was needed, which allowed activity snacks to conveniently fit into their lives.

I think it [Snacktivity] would be a good idea for anybody of every age, yeah. Patient 27

[…] As I say, it’s stuff that you can fit in, it’s, it’s not… as long as it’s not mandatory, then you sort of… you’ve got a chance to be able to fit it in anywhere. Patient 3

#### Easy or Manageable

About half of participants viewed Snacktivity as easy to do, with a few others viewing it as ‘manageable’, referring to Snacktivity as a more realistic goal to reach because it fitted into their daily routine, that it took account of their capabilities and the demands on their time.

So an easy way to, to add in movement, it’s great. I think it’s a really good idea. Patient 4

It’s [Snacktivity’s] easier than sort of saying, ‘Oh you must go out and have a run or take a walk or … or go to the gym’. I think it’s much more manageable. Patient 23

#### Health Benefits

About a third of participants viewed Snacktivity as a method to maintain their mobility and stay physically active, and a third viewed Snacktivity as beneficial to health, particularly in reducing the time spent sedentary each day.

Well I think sort of what I’ve been saying really, it’s just keeping your muscles going and, using the, a few calories up and it’s… and keeping your joints active as well, so that your whole body’s you know keeping, keeping in trim. Patient 2

I like it [Snacktivity] personally. I don’t know whether it’ll actually make, you know … help to meet what the NHS recommend. But it certainly is making me, you know more active in a little way, so where I would normally sit at work all day…I now do break it up with little Snacktivities. Patient 20

#### Killing Two Birds with One Stone

Around half of participants suggested Snacktivity had the benefit of ‘killing two birds with one stone’, as many activity snacks could be undertaken at the same time as doing other tasks such as when completing errands, rather than having to compete for time against other daily activities and priorities. Some participants perceived Snacktivity as requiring little, or often no time, for the very reason that it can be achieved with other activities each day.

I absolutely hate [housework], but, again I wanted to get it done as soon as possible, in the least amount of time possible because I hate it! [...] But then when I realised, okay, I can count this as an exercise activity! So I thought ‘Okay, brilliant! Staff 4

#### Changes in Perspective

Most participants said that Snacktivity gave them a new awareness that more physical activity could be added into their day, and they were now more mindful of these opportunities. Some participants said Snacktivity increased their motivation for physical activity.

It [Snacktivity] does make you think… that yes, we should be doing a bit more when… and we’ve got the opportunity to do it as well. [...] It’s just encouraging… movement, you know, instead of sitting down can I get up and do something and stay, standing whilst I’m doing it? Patient 7

I found it helped me to re-motivate me actually the last few days. Patient 11

Some participants considered Snacktivity novel, viewing it as either giving them new ideas to try out, or providing a new framework for activity snacks they had already been completing, but did not perceive as ‘snacks’. Some participants also believed that Snacktivity challenged ‘conventional wisdom’, that long bouts of vigorous physical activity or exercise are needed to experience any impactful benefits to health, changing to now viewing and understanding that any physical activity is beneficial for health, even short ‘bites’ at a lower intensity.

The arm raises and then, the press-ups against the stairs, I hadn’t thought of that, that before, so … and I thought, ‘Ooh I could actually do that’, and I do up and down the stairs a lot, I have to, and I’m quite pleased I’ve got stairs! Patient 9

Anything that, breaks down the sort of, barriers for doing anything, kind of, is a good idea and anything that you know… This idea isn’t it? That you don’t do everything, there’s no point doing anything. […] It [Snacktivity] begins to break down that, concept that… and approaches it the other way that anything you do is good, so you might as well do… that it’s better to be doing something than nothing. Patient 10

#### Snacktivity Is About Food

When initially receiving the study documentation, about one quarter of participants had initially thought the Snacktivity idea to be about food or diet or found that others initially assumed this when describing idea to them.

Snacktivity, when I first got when I first saw that [name], I thought that meant eating! Patient 8

I just tried to explain it to a colleague because a colleague had heard of the study said ‘I don’t want to do that because I don’t eat snacks. Staff 3

Some participants understood Snacktivity to be free to do. A few participants initially thought that most people would already be doing the snacks, but then realised this was not the case. Some thought that Snacktivity would also help keep their mind active.

At first I was very sceptical [of the Snacktivity idea] because I thought that most people would actually already be doing things like that anyway. [...] They obviously don’t. And I think it would be a good idea to remind you to do it. Patient 3

I think, it’s [Snacktivity’s], a really good idea. […] especially now when we’re all, locked down and we’re all at, at home, it’s… and we don’t have access to our gyms and… you know not doing the things that we used to do. Patient 4

#### Feelings About the Snacktivity Idea

All participants felt positive towards the Snacktivity idea, or some aspect of it, with almost all describing it as a ‘good’, ‘great’ or an ‘excellent’ idea.

I think it’s [Snacktivity’s] a brilliant idea… I really do, I mean because it’s stuff that… anybody can do, without interfering with anything […] …I think it’s a damn good idea. I think it’s a really, really good idea. Patient 27

I, I think it’s [Snacktivity’s], you know, particularly as you get, older or you do a sedentary job, I think it’s… very, vital. Patient 2

### Likes or Dislikes About Snacktivity

Around half of participants stated that there was nothing that they particularly disliked about Snacktivity, although a few listed dislikes in relation to specific activity snacks. About a quarter liked that Snacktivity was easy, could be tailored to individuals; personal circumstances, was ‘bite size’, add physical activity into their life, offered a range of activities to choose from, and fitted into their everyday routine. A few participants liked that Snacktivity was free, that it allowed them to be physically active, whilst also being free from others’ judgement, it could be completed any time of the day; it motivates and could be completed in any location, without need for equipment or a change of clothing.

Snacktivity helped me to just kind of like well you can work with it you know! But at your own pace! So you know I’m just really, really glad, really grateful for that. Staff 4

I like it [Snacktivity] because, you haven’t got to go to a class or anything, you can, you can do it within the day. Whatever you’re doing in that day, there’s always something on there where you can actually practice, some of the snacktivities. Patient 22

Participants’ dislikes of Snacktivity were more diverse compared to elements that they liked. These dislikes typically related to specific activity snacks making them look or feel uncomfortable (e.g., lunges by the printer at work and skipping). A few participants felt that some of the activities and some images in the Snacktivity picture board could be more inclusive, particularly for older aged adults. Two participants disliked the term Snacktivity, which they assumed was related to making changes to their diet in some way.

I think it’s a good idea. I think that, maybe … maybe some of it could be more targeted to… perhaps age groups and, things like that, people who are retired and pensioners wouldn’t be taking the children they might not have a skipping rope, they wouldn’t be playing with their children and things like that. Patient 3

### Perceptions of How Others Would Respond to Snacktivity

#### Friends and Family

Most participants thought that their friends and family would think that Snacktivity was a good idea, and many had received positive feedback when discussing their involvement in this study. Some participants felt that the response from their family/friends to the Snacktivity concept would be mixed. Two participants felt their family/friends would think Snacktivity was ‘tame’ or the novelty (as it would be with any new approach or physical activity routine) would diminish over time. One participant thought their friends would ignore it.

[..] because I’ve got four children, I’ve told them all about, what I’ve been doing and you know what have you. And I’ve explained the idea to them, because they’re all like early thirties now, and they, you know, they think it’s a great idea. Because… I think they appreciate… the need for some level of exercise in everyone. I really do. Patient 5

### Government Recommendation to Promote Snacktivity

When asked about the likely response from the public to the government hypothetically recommending daily Snacktivity, participants expressed a variety of views. About half of participants thought that Snacktivity would be well received by the public, while others gave responses that referred to the ‘nanny state’ concept, that is, the government interfering in aspects of individuals’ lives that it should not. Most of these participants thought that only *some people*, *not all*, would resist the message.

NT: If the government issued guidance encouraging the public to take part in Snacktivity every day, how do you think the public would respond?

P: …Mmm, well I think you’d get a division really. Because, half of them would say ‘Why should the government tell me what to do, yeah? Patient 25

I think for older people it’d have a good response. I think for kid… they’re active anyway, aren’t they? So I think for older people, retired people, people who aren’t very active and need a little bit of exercise, I think, it would be a good response. Patient 27

Some participants appreciated the difficulty of getting a large-scale campaign to change people’s health behaviours and were concerned about how the message would be conveyed.

I think my only caution would be, is that people will view it instead of something [exercise] […] some people will feel that actually it’s not good enough. So anyway when I talked it through with my husband, he’s like ‘No, no, don’t want that. [...] Why would you [only] do ten minutes? Ten minutes isn’t going to do anything for you. ’So I think there is something there about building up the narrative around that, I think that’s going to be incredibly important. Staff 3

### Facilitators to Physical Activity

#### Snacktivity Increases Physical Activity

Most participants reported that their physical activity had increased while doing Snacktivity, either modestly or by a substantial amount. Participants who had not experienced this, had nevertheless thought that Snacktivity had made them more focused on, or aware of, opportunities for physical activity.

Definitely! Definitely it’s [Snacktivity], it’s helped. Because before, you’re not really… you don’t have to do anything, you’re just slotting it into something you already do […] so definitely… definitely increased [my physical activity]. Patient 4

#### Technology and Reminders

Most participants suggested or reported that having a physical activity mobile app or tracker could provide information to allow people to better track their physical activity and this would encourage them to do more. A few participants thought timed reminders (e.g., texts and notifications) would encourage them to do more physical activity.

I don’t think, I was really aware… before I got one of these how, little steps I could do some days. You know in a busy workday where you’re stuck at your laptop all day, I don’t think I quite realised how inactive I was… till I started to see. Patient 11

NT: Are there any other strategies that you think might help you become more active?

P: Text reminder, yeah, possibly that, that would… would have an impact. Patient 10

#### Encouragement from Others, Accountability, and Goals.

Around a third thought that encouragement from other people in various ways would increase their physical activity: texts from people they know; competing with their spouse; informal support from family and friends; encouragement from groups of strangers or friends, face-to-face, online, or through mobile phone apps; or exercising with friends. Around a third thought that some form of accountability to check on their activity, such as a walking buddy, a personal trainer, or a follow-up call, would help them become more active.

#### Feedback and Progress

The most reported reason for maintaining physical activity was enjoying the activity itself (around a third). Some reported liking seeing results from their physical activity or experiencing a sense of progress from seeing improved strength, balance, or becoming less out-of-breath. Around a quarter reported that Snacktivity fitted into their schedule.

NT: Is there anything that you think helps you keep sticking to [physical activity]?

P: Just that I enjoy it. I think the thing with any activity is if you enjoy it. Patient 23

#### Making Snacktivity a Habit

Most participants believed it was important to make Snacktivity a habit to ensure they did it. Most participants reported that Snacktivity was easy to remember, with some reporting that it was not always easy to remember when their routine changed.

NT: Did you find anything stopped you from doing Snacktivity each day?

P: Did it stop… No, apart from the first, first day when I did the whole day and I, I forgot! And then I had to quickly squeeze it in before bedtime! Patient 4

### Barriers to Snacktivity

The most common reported barrier to undertaking Snacktivity was forgetting to do it, as reported by a few participants, and some thought that medical conditions, or being ill, could be a barrier to both Snacktivity and specific activity snacks. A few participants thought that there were no barriers to Snacktivity per se, but some suggested barriers to specific activity snacks. Work and prioritising other things were reported by some participants as barriers to achieving Snacktivity. Time, being busy, and the workplace culture were reported as barriers by two participants.

The culture in my workplace, everyone usually stays inside, indoors and you know, the most you do is make a round of tea. [...] You don’t really go up and down, like you stay in your own floor. So it’s just, there isn’t a culture of, oh ‘Let’s go out for lunch and walk around or go to’ … there isn’t any parks around, so you know it’s not like there’s a culture of moving around. Staff 2

### Suggestions for Improving the Snacktivity Concept

Participants made several suggestions for how the Snacktivity concept could be developed. A few participants suggested that it may be useful for the public to be able to identify which activity snacks are more vigorous than others on the picture board. One participant suggested distinguishing between moderate and vigorous intensity activity snacks, while another suggested developing Snacktivity workouts that consisted of several different snacks of activity that people could work through. Another participant suggested offering short and longer duration activity snacks (big snacks and little snacks).

## Discussion

This study explored the views of the public about Snacktivity as a novel approach to promoting physical activity in the population. Whilst participants recognised that the Snacktivity approach had merits, they did raise several potential barriers to implementation. Findings demonstrated that participants understood and liked the concept of Snacktivity and viewed the idea of ‘bite sized activity snacks as an acceptable approach to promoting physical activity in the population. Participants discussed that Snacktivity allowed them to feel a sense of achievement because it was easy to achieve. Several suggestions were proposed by participants regarding the development of the Snacktivity approach, to ensure it fits with the needs, routines, and lifestyles of the population.

### Interpretation of the Findings

All participants understood the concept of Snacktivity. The idea that Snacktivity ‘killed two birds with one stone’, that it provided flexibility, and could be achieved while completing daily tasks were particularly appreciated by participants. Many participants commented that integrating Snacktivity into their lives (prior to their interview) had given them a new awareness of their ability to achieve more activity (Snacktivity), and they felt motivated by these new opportunities. Motivation is considered a persuasive factor toward the willingness to change, and the attributes of efficiency and flexibility may in turn increase people’s motivation to engage in physical activity; this is critical given studies show that the main reason for inactivity is perceived lack of time [27]. Historically, physical activity has often been perceived by the public as an obligatory sport-based behaviour requiring considerable time and energy to experience health benefits [28]. Snacktivity offers an opportunity to move towards the perception that physical activity can be convenient and self-directed, and that it can be undertaken by most people, in any location and/or time of day.

Unsurprisingly participants disliked activity snacks which they perceived as embarrassing or uncomfortable to do. It is also interesting to note that skipping was not liked by participants — an activity that requires equipment, is skill-based, high intensity, and requires physical coordination. Activity snacks that have these elements may be less desirable. Participants appreciated that Snacktivity gave them a different perspective, a new way of thinking about how to be physically active and they liked the broad range of activities that the Snacktivity approach offered them. Psychological theories (self-determination theory and self-regulation theory) also emphasise the importance of focusing on control or related constructs in the behaviour change process [29–31]. Self-determination theory points to importance of autonomy as a condition that fosters volitional and high-quality forms of motivation and engagement for activities, highlighting the importance of offering choice of actions when aiming to foster health behaviour change.

Snacktivity seeks to combine concepts and behaviours to translate physical activity messaging in a novel format that is innovative and attractive to the public. When first approached to take part in this study, several participants initially thought they were being invited to take part in a study about food and eating. The term ‘snacks’ or ‘snacking’ have traditionally been associated with eating, a behaviour that is detrimental to health. For Snacktivity to resonate, with the public, the concept of ‘snacking’ will need to be realigned in the minds of the public, from being an unhealthy eating behaviour that should be avoided, to also being a health behaviour that is in fact good for health and encouraged.

Consistent with previous studies, typical barriers to physical activity included having other interests, lack of motivation, health limitations, work, and lack of time [11, 16]. Regarding Snacktivity specifically, a somewhat common barrier was forgetting to do Snacktivity. Several of these barriers point towards issues with participants being able to regularly schedule Snacktivity into daily life, which may be linked with their self-regulatory self-efficacy and/or motivation to do so [32]. Participants suggested that such barriers could be addressed by providing people with prompt reminders, for example, by using phone reminders and physical activity trackers that alerted people when they have been sedentary for too long. This suggestion is worthy of consideration given that studies have reported mobile phone Apps and technology can be effective in facilitating behaviour change [33]. Finding the motivation to engage with Snacktivity was also raised by participants as a potential barrier. No physical activity strategy or intervention will be effective if the public do not engage, with it. Motivation is core to any behaviour change strategy and for Snacktivity to be effective it will be critical to consider how motivation for frequent engagement with Snacktivity can be promoted in the public over the long-term, to maximise their efficacy beliefs, that they are able to regularly include Snacktivity within daily life. Related to this, it was interesting that participants felt that for Snacktivity to be successful, it needed to become a habit, a view consistent with the habit formation model [34, 35]. Strategies to foster habit formation and self-regulatory self-efficacy for Snacktivity, such as reminders, cues, nudges, and tools for self-monitoring and subsequent reflection, planning opportunities, a focus on executing new behaviours, engaging in activity snacks that are easy to complete which minimise demands on individuals’ mental resources, would appear to be a worthwhile focus for the development of the Snacktivity approach [36].

There was an assumption amongst some participants that physical activity is only beneficial for health if undertaken in long(er) bouts. This finding is consistent with previous physical activity guidance, which had indicated that physical activity needed to be at least 10 min in duration to benefit health, but updated guidance does now include a focus on any activity is better than none, and more is better still [1, 5]. Participants in other qualitative studies have also reported that the message of ‘some is good, more is better’ struck the right tone with them [37]. Brawley and Latimer [4] have discussed the importance of packaging guidelines into messages that offer specific content, are based on scientific recommendations, and encourage specific groups to meet the guidelines. The translation of physical activity guidelines needs to be widely disseminated, and the Snacktivity message is one means by which this could be achieved.

### Implications

Physical activity promotion has often been centred around encouraging single activities, such as running, walking, swimming, and tennis. In contrast, Snacktivity seeks to offer the public the opportunity to engage in range of activities, or to ‘pick and mix’ activity snacks, with a view to making physical activity more appealing, varied, sustainable and enjoyable to the population, regardless of ability, age, physical status, socio-economic background, and environmental context. It will be important to ensure that Snacktivity involves all these tenets and emotions to maximise engagement and adherence. Moreover, there is evidence that the public would prefer language within guidance that is ‘snappy’, ‘chatty’, and ‘encouraging’ and ‘which uses humour’ [37]; Snacktivity has the ability to incorporate these preferences.

### Strengths and Weakness

This study has several merits of note. To our knowledge, no previous studies have explored the views of the public about the Snacktivity concept, and therefore, this study adds new knowledge. Physically inactive adults from a range of socioeconomic backgrounds and ethnicities were recruited. Specifically, 26% of the study sample were from a non-White ethnic background, a higher rate than recorded for England and Wales (16.7% of the population) [38]. A comprehensive method to data analysis was adopted. Data analysis was facilitated by four researchers from differing backgrounds and perspectives, ensuring theme development and interpretation of data was considered from several viewpoints and experiences. This study adds value to knowledge because findings will contribute to the development of future interventions, and it highlights potential for changes in the way in which the public conceptualise physical activity. This study also has some limitations. Of those approached to participate, less than half agreed and it may be that those recruited were motivated ‘innovators’ with a more positive view of physical activity than those who declined. A third of participants were retired, and findings may align more closely with the views of older adults than their younger counterparts.

## Conclusion

Guidance must lead to advice that has the best chance of preserving and promoting health. Guidance for physical activity needs to be creative, flexible, and agile; Snacktivity may offer the opportunity to fulfil these needs. Our study has offered some new insights about the Snacktivity approach to promoting physical activity in the population, and these findings now need to be translated into testing real world interventions that can prompt the integration of Snacktivity into the daily lives of the population.

## Supplementary Information

Below is the link to the electronic supplementary material.

Supplementary file1 (PDF 6057 KB)

## Data Availability

The datasets used and analysed during this study are available from corresponding author on reasonable request. Access to anonymised data may be granted following review of the request. Exclusive use will be retained until the publication of major outputs from this research programme.

## Declarations

### Disclaimer

The views expressed are those of the author(s) and not necessarily those of the NHS, the NIHR, or the Department of Health and Social Care.
