# Supplementary material for: Snacktivity™ to Promote Physical Activity: a Qualitative Study
Source: Int J Behav Med. 2021 Nov 15;29(5):553–64. doi: 10.1007/s12529-021-10040-y (PMC8592280; doi:10.1007/s12529-021-10040-y)
Supplement: Supplementary file 1 — Supplementary file1 (PDF 6057 KB) [file 12529_2021_10040_MOESM1_ESM.pdf]

Snacktivity

Picture Booklet

Activity snacks you can do while at work

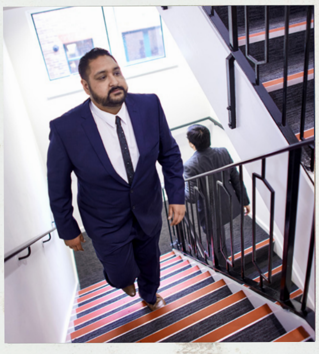

1 Take the stairs instead of the lift or escalator

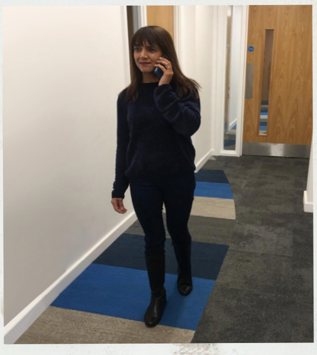

2 Move away from your desk and walk whilst taking calls

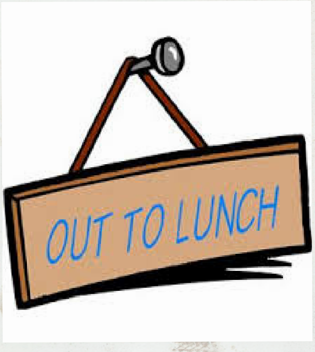

3 Go for a brisk walk at lunchtime

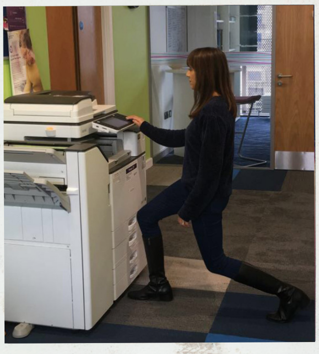

4 Do lunges at work

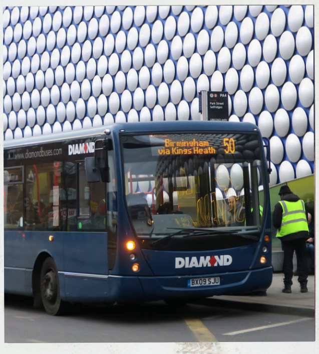

5 Get off the bus one stop earlier & walk

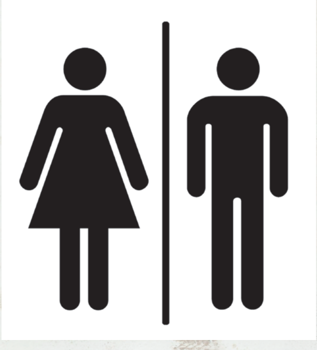

6 Use a toilet that is the furthest away/on another floor

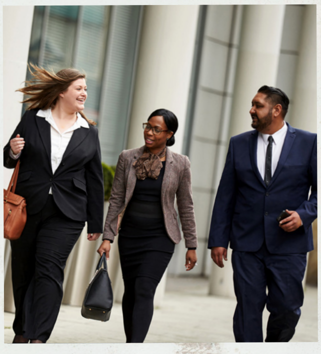

7 Have a walking meeting with colleagues

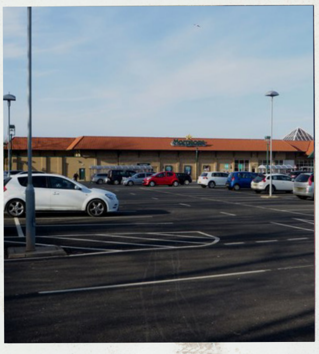

8 Park your car further away and walk

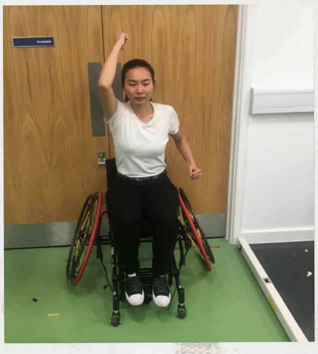

9 Do arm raises whilst seated

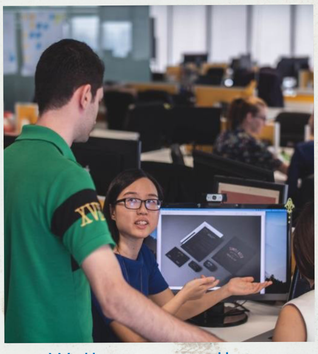

10 Walk over to talk to colleagues instead of using the phone or email

Activity snacks you can do at home and in your leisure time

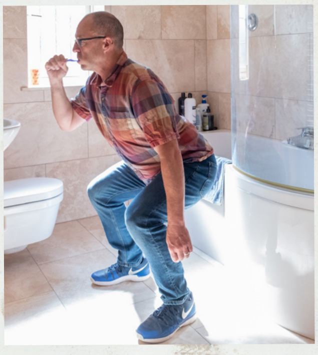
11 Do squats whilst brushing your teeth

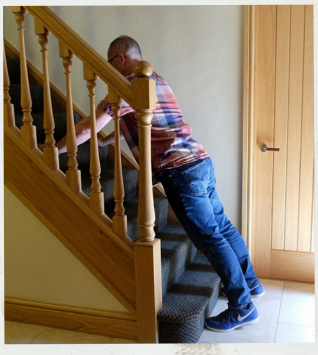
12 Do press-ups against the stairs

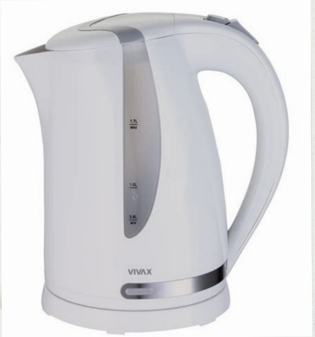
13 Keep moving whilst you wait for the kettle to boil

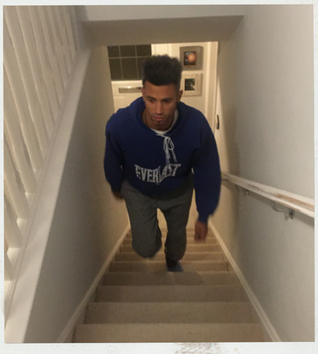
14 Walk up and down the stairs multiple times

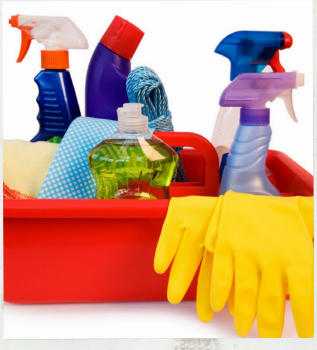
15 Do some housework

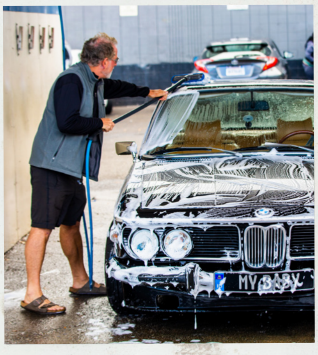
16 Wash your car

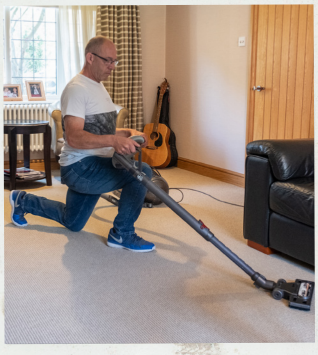
17 Do lunges whilst you vacuum the house

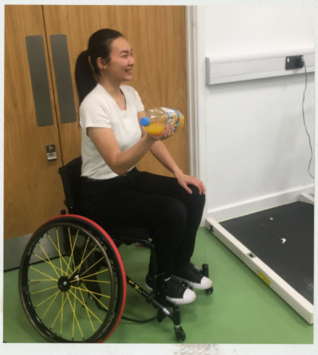
18 Do bicep curls with tins/a bottle whilst seated

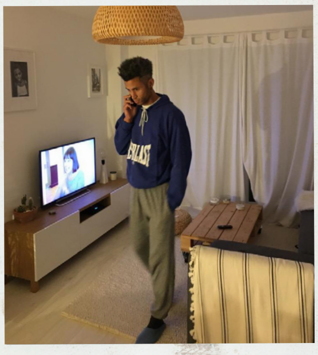
19 Walk whilst talking on the phone

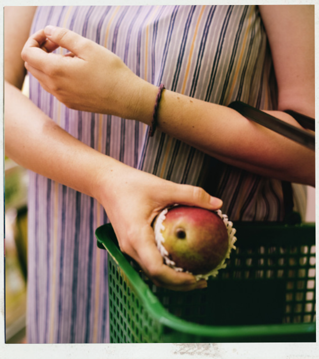
20 Use a basket whilst shopping instead of a trolley

Please turn over

Activity snacks you can do at home and in your leisure time

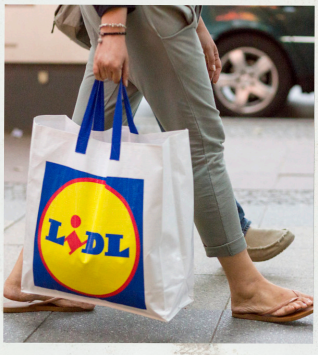

21 Walk/ run/ cycle to the local shops

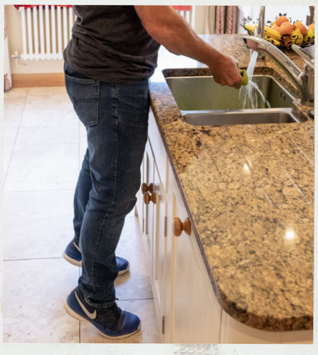

22 Do calf raises while washing the dishes

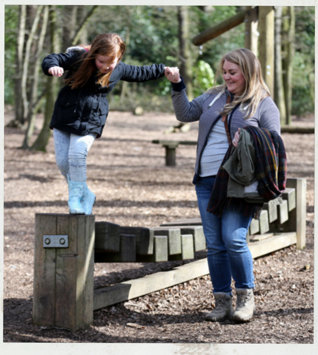

23 Play with your children at the park

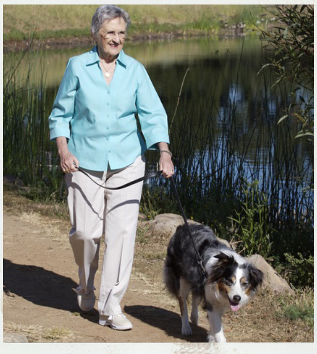

24 Take your dog for an extra brisk walk

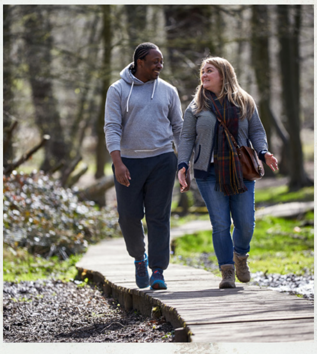

25 Go for a brisk walk around your local park

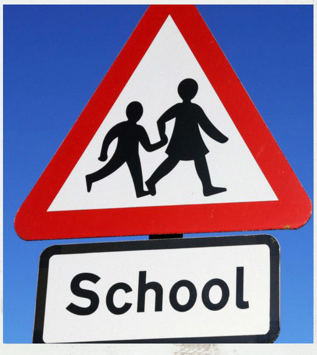

26 Walk to drop/pick your children from school

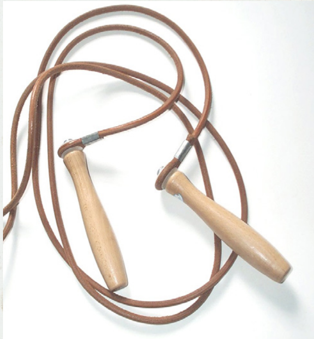

27 Take up skipping

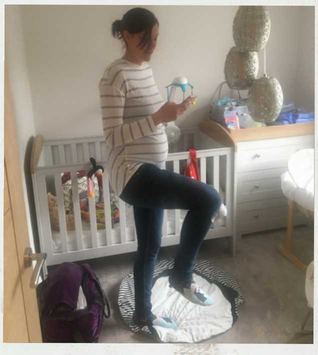

28 March on the spot

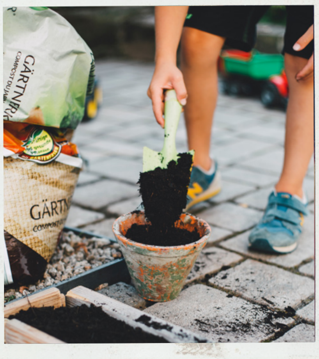

29 Do some gardening

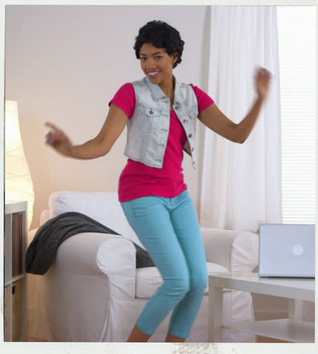

30 Dance around the living room/kitchen
